# Reproductive Toxicity of Immune Checkpoint Inhibitors in Triple-Negative Breast Cancer: A Case Report with a Literature Review

**DOI:** 10.3390/diseases14020051

**Published:** 2026-01-30

**Authors:** Cristina Tanase-Damian, Nicoleta Zenovia Antone, Diana Loreta Paun, Ioan Tanase, Patriciu Andrei Achimaș-Cadariu

**Affiliations:** 1 Faculty of Medicine, “Iuliu Hatieganu” University of Medicine and Pharmacy, Victor Babes 8, 400347 Cluj Napoca, Romania; nicopopanton@yahoo.com (N.Z.A.); patrick.achimas@hotmail.com (P.A.A.-C.); 2 Department of Oncology, The Oncology Institute “Prof. Dr. Ion Chiricuta”, Republicii 34-36, 400015 Cluj Napoca, Romania; 3 Department of Endocrinology, “Carol Davila” University of Medicine and Pharmacy, Eroii Sanitari 8, 050474 Bucharest, Romania; diana.paun@umfcd.ro; 4 Department of Surgery, “Carol Davila” University of Medicine and Pharmacy, Eroii Sanitari 8, 050474 Bucharest, Romania; ioan.tanase@umfcd.ro; 5 Department of Gynecology-Oncology, The Oncology Institute “Prof. Dr. Ion Chiricuta”, Republicii 34-36, 400015 Cluj Napoca, Romania

**Keywords:** triple-negative breast cancer, immune checkpoint inhibitors, reproductive toxicity, fertility, hypogonadism, oncofertility, premature ovarian insufficiency

## Abstract

Triple-negative breast cancer (TNBC) is an aggressive malignancy that disproportionately affects young women. The integration of immune checkpoint inhibitors (ICIs) has significantly improved outcomes in both early-stage and metastatic TNBC, shifting attention toward long-term survivorship issues, particularly endocrine function and fertility. However, the reproductive safety profile of ICIs remains insufficiently characterized. This narrative review synthesizes current preclinical and clinical evidence on ICI-associated reproductive toxicity, focusing on both direct immune-mediated gonadal injury and indirect disruption of the hypothalamic–pituitary–gonadal axis. Experimental models consistently demonstrate immune cell infiltration of ovarian and testicular tissue, cytokine-driven inflammatory cascades, follicular atresia, impaired spermatogenesis, and altered steroidogenesis following PD-1/PD-L1 and CTLA-4 blockade. Emerging clinical data report cases of immune-related orchitis, azoospermia, testosterone deficiency, diminished ovarian reserve, and premature ovarian insufficiency. Secondary hypogonadism due to immune-mediated hypophysitis represents an additional and frequently underdiagnosed mechanism. We further discuss the oncofertility challenges faced by young patients with TNBC treated with chemoimmunotherapy, emphasizing the uncertainty of fertility risk stratification and the importance of early fertility counseling and individualized fertility preservation strategies. To illustrate the potential clinical impact, we present the case of a 34-year-old nulliparous woman who developed premature ovarian insufficiency two years after neoadjuvant chemoimmunotherapy including atezolizumab, despite ovarian suppression. In conclusion, while ICIs have transformed the therapeutic landscape of TNBC, their potential long-term impact on reproductive and endocrine health represents a clinically significant concern. A precautionary, multidisciplinary oncofertility approach and prospective clinical registries are essential to define the true incidence and mechanisms of ICI-associated reproductive toxicity.

## 1. Introduction

Triple-negative breast cancer (TNBC) accounts for approximately 12% to 17% of all breast cancers [1] with a high degree of aggressiveness, and compared with other breast cancer types, TNBC disproportionately affects women younger than 40  years old [2]. It is defined by the absence of estrogen receptor (ER), progesterone receptor (PR), and human-epidermal growth factor receptor 2 (HER2) expression, which precludes the use of endocrine or HER2-targeted therapies [3,4]. Until recently, the cornerstone of oncological treatment for both localized and metastatic disease, has historically been aggressive chemotherapy, but the results were poor, with a median survival less than 18 months [5,6,7].

The fast progress in cancer treatment regimens has redirected focus from traditional cytotoxic therapies to a new era of long-lasting, tailored cancer care such as immunotherapy [8].

Despite these therapeutic benefits, little is known about the potential reproductive toxicity of immune checkpoint inhibitors. Unlike chemotherapy, where gonadotoxicity is well documented, the impact of ICIs on fertility and gonadal reserve remains largely unexplored. The available literature on ICI-associated reproductive toxicity continues to be fragmented, consisting mainly of preclinical studies, pharmacovigilance analyses, small retrospective cohorts, and isolated case reports. While these data suggest that immune-mediated reproductive injury is biologically plausible, the true incidence, severity, reversibility, and long-term consequences remain unknown. This knowledge gap poses a significant challenge for clinicians counseling young women with TNBC regarding fertility preservation and family planning.

Although international guidelines (ASCO, ESMO, ESHRE) emphasize early fertility counseling before systemic therapy, they provide limited recommendations specific to patients receiving ICIs [9,10], as is illustrated in Table 1.

Against this backdrop, the aim of this review is to critically synthesize current preclinical and clinical evidence on the reproductive toxicity of immune checkpoint inhibitors, with a specific focus on triple-negative breast cancer. We analyze the mechanistic basis of immune-mediated gonadal injury and endocrine disruption, summarize reported male and female reproductive adverse events, discuss implications for oncofertility counseling and fertility preservation, and highlight unmet research needs. In addition, we present an illustrative clinical case of premature ovarian insufficiency following chemoimmunotherapy including atezolizumab, underscoring the potential real-world impact of these mechanisms.

## 2. Methods

### 2.1. Study Design

This manuscript was conducted as a narrative review aiming to synthesize current evidence regarding the potential reproductive and endocrine toxicity of immune checkpoint inhibitors (ICIs). Given the emerging nature of this field and the limited availability of prospective clinical data, a narrative approach was considered the most appropriate to integrate mechanistic, clinical, and translational evidence.

### 2.2. Literature Search Strategy

A literature search was performed using PubMed/MEDLINE, Embase, and Web of Science to identify relevant publications up to January 2025. Search terms included combinations of immune checkpoint inhibitors, immunotherapy, triple-negative breast cancer, fertility, ovarian function, hypogonadism, reproductive toxicity, endocrine toxicity, premature ovarian insufficiency, and oncofertility. In addition, reference lists of key articles and relevant international guidelines were manually screened to identify additional relevant publications.

### 2.3. Study Selection and Evidence Synthesis

Eligible sources included preclinical studies, pharmacovigilance analyses, retrospective clinical studies, case series, case reports, and international clinical guidelines addressing reproductive or endocrine outcomes associated with immune checkpoint inhibitors. Given the scarcity of high-level evidence, no restrictions were placed on study design.

### 2.4. Clinical Case Illustration

A clinical case was included to illustrate a plausible real-world scenario of ovarian dysfunction following exposure to multiple potentially gonadotoxic treatments. The case is presented for illustrative and hypothesis-generating purposes only and is not intended to establish causality or generalizability.

Importantly, within the setting of triple-negative breast cancer, immune checkpoint inhibitors are almost invariably administered in combination with cytotoxic chemotherapy, an already well-established cause of gonadotoxicity. This therapeutic overlap represents a major confounding factor when evaluating reproductive and endocrine outcomes. As a result, disentangling immune-mediated ovarian effects from chemotherapy-induced gonadal damage remains extremely challenging based on currently available clinical data. Throughout this review, reported reproductive toxicities are therefore interpreted with caution, and associations are discussed within the context of combined treatment exposure rather than attributed to immune checkpoint inhibitors alone.

## 3. The Gonadal Toxicity of Immune Checkpoint Inhibitors

### 3.1. The Direct Effect on Testicular Function

ICIs can have a direct effect on the endocrine system, including the gonads, which can lead to primary hypogonadism and infertility. Clinically, this translates into a reduced or impaired production of viable oocytes or spermatozoa and a fertility issue. Primary hypogonadism is biochemically defined by a reduction in circulating sex hormone levels, such as testosterone or estradiol, coincident with an increase in the secretion of gonadotropins—luteinizing hormone (LH) and follicle-stimulating hormone (FSH)—as a result of disrupted gonadal activity. In women, there could also be a reduction in anti-Müllerian hormone concentration, with a low ovarian reserve and reproductive consequences [11].

However, the actual occurrence of primary hypogonadism is unknown [12].

Only one case of primary hypogonadism was identified in a recent analysis of Vigi Base [13], but this may be due to underassessment and underreporting of these patients.

Most existing preclinical and clinical research on immune checkpoint inhibitors (ICIs) has concentrated on tumor regression and identifying predictive biomarkers of therapeutic response. However, comparatively limited focus has been placed on how ICIs influence immune cell dynamics and cytokine levels in patients. Despite this gap, growing evidence shows that ICIs cause notable shifts in circulating immune cell profiles and promote an increase in systemic pro-inflammatory cytokines, indicating that their impact extends beyond direct tumor targeting [14].

Systemic immune activation induced by ICIs is accompanied by sustained increases in circulating pro-inflammatory cytokines, including interferon-γ (IFN-γ), tumor necrosis factor-α (TNF-α), interleukin-1β (IL-1β) [15]. PD-L1 blockade has been shown to significantly improve circulating IFN-γ, IL-6, and IL-18 [16,17]. Fluctuations in the population and functional activity of circulating immune cells, in conjunction with altered cytokine profiles, may impair physiological processes within the ovaries or testes.

Although the majority of oncofertility research has historically focused on women, emerging evidence indicates that ICIs may also adversely affect male reproductive function through both direct testicular injury and secondary endocrine disruption. While large prospective studies are lacking, data derived from case reports, retrospective cohorts, autopsy series, and small cross-sectional studies collectively suggest that male gonadal toxicity is a clinically relevant and potentially irreversible complication of immunotherapy.

Animal studies have demonstrated that ipilimumab treatment in monkeys can reduce testicular weight, though without noticeable alterations in sperm tissue structure [13]. Conversely, pembrolizumab exposure in rats has been associated with both structural damage to the testes and impaired sperm quality [16]. Additionally, various clinical reports and case studies have indicated that immune checkpoint inhibitors (ICIs) may directly affect male reproductive function.

The most direct clinical expression of immune-mediated testicular injury under ICI therapy is the occurrence of orchitis and epididymo-orchitis. Two well-documented case reports describe acute inflammatory testicular syndromes developing shortly after the initiation of PD-1 and/or CTLA-4 blockade [18,19]. In one notable case, a middle-aged man with a metastatic melanoma developed acute bilateral orchitis two weeks after the combination of ipilimumab and nivolumab therapy. Laboratory evaluation revealed markedly suppressed testosterone levels with a compensatory elevation of luteinizing hormone, consistent with the primary hypogonadism. The rapid onset of symptoms, the exclusion of infectious causes and the spontaneous resolution without antimicrobial therapy strongly support an immune-mediated etiology. Although clinical recovery occurred, the long-term impact on spermatogenesis could not be evaluated due to the patient’s refusal to undergo semen analysis [18].

Nonetheless, this case underscores the potential fertility implications associated with immune checkpoint inhibitors [18].

The second report described severe anti-PD-1-induced epididymo-orchitis associated with immune-mediated encephalitis, which responded favorably to corticosteroid therapy [19]. These rare but instructive cases provide direct clinical proof that ICIs can trigger organ-specific autoimmune inflammation within the male reproductive tract.

Importantly, even transient inflammatory damage to testicular tissue may have lasting reproductive consequences. Urogenital inflammatory diseases, including infectious orchitis and epididymo-orchitis, have been independently associated with impaired sperm parameters, increased DNA-fragmentation, and subfertility in non-oncologic populations [20,21]. By analogy, immune-mediated testicular inflammation during ICIs therapy may pose a similar fertility risk.

Beyond acute inflammatory events, more insidious forms of spermatogenic failure have also been reported. One striking case described a previously normozoospermic man who developed complete azoospermia two years after combined anti–PD-1 and anti–CTLA-4 therapy for metastatic melanoma [22]. The testicular sperm extraction failed to retrieve viable spermatozoa, and histopathological analysis showed Sertoli cell-only pathology [22].

This observation highlights a critical aspect of ICI-related gonadal toxicity: reproductive failure may occur long after treatment completion, potentially escaping early clinical detection. The delayed onset further supports an immune-mediated mechanism characterized by progressive damage to the germinal epithelium rather than acute cytotoxic injury.

The strongest histopathological evidence linking ICIs to impaired spermatogenesis derives from a retrospective autopsy study of patients with metastatic melanoma treated with immunotherapy [23]. In this series, testicular tissue from men exposed to ICIs was compared with that of age-matched controls who had not received immunotherapy. Remarkably, more than 80% of ICI-treated patients exhibited compromised spermatogenesis, including hypospermatogenesis, focal active spermatogenesis, and Sertoli cell-only syndrome [23]. In contrast, only a minority of controls showed impaired spermatogenesis [23].

Although limited by retrospective design and the inherent selection bias to autopsy-based studies, these data provide compelling structural evidence of treatment-associated spermatogenic failure at tissue level.

Functional fertility data in men treated with ICIs remain scarce. A small cross-sectional pilot study evaluated semen parameters in men undergoing or previously exposed to immunotherapy [24]. The majority of patients demonstrated normal spermiograms; however, a subset exhibited oligoasthenoteratozoospermia or azoospermia. Importantly, several of these patients had significant confounding factors, including prior chemotherapy, radiotherapy, bacterial orchitis, and chronic alcohol abuse.

Nevertheless, one patient with documented normal baseline semen parameters prior to ICI exposure later developed azoospermia associated with inflammatory testicular infiltrates, strongly suggesting a direct immunotherapy-related effect. The very limited availability of baseline semen analyses remains a major obstacle in establishing causality [24].

Another retrospective study reported that nearly 70% of men receiving anti-CTLA-4 and/or anti-PD-1 had low serum testosterone levels during treatment [25]. Symptoms like fatigue, sexual dysfunction, and reduced libido were frequently reported, yet only a small proportion of affected patients received testosterone replacement therapy [25]. However, interpretation of these findings is complicated by several confounders including lack of consistent baseline hormonal evaluation, variable timing of testosterone measurement, presence of systemic illness, concomitant therapies [25]. Despite these limitations, the possibility of ICI-induced Leydig cell dysfunction, whether primary or secondary to pituitary involvement, remains biologically plausible and clinically relevant.

Although most male patients treated with ICIs do not develop overt gonadal toxicity, the available evidence supports the existence of a non-negligible risk of irreversible spermatogenic failure and androgen deficiency in a subset of patients. This risk is particularly relevant for young cancer survivors with long life expectancy following immunotherapy.

From a clinical standpoint, these observations hold several substantial implications:

- Baseline fertility assessment, including semen analysis and reproductive hormone profiling, should be considered in men of reproductive age prior to initiating ICIs, especially when used in curative settings.
- Sperm cryopreservation should be systematically discussed before starting immunotherapy, particularly in patients anticipating prolonged treatment durations or combination regimens.
- Long-term andrological follow-up is essential, as gonadal dysfunction may emerge months or years after treatment completion.

Importantly, current oncologic guidelines emphasize the importance of fertility preservation prior to gonadotoxic chemotherapy but remain largely nonspecific regarding ICIs. As immunotherapy increasingly integrates into early-stage cancer management, this guidance gap warrants urgent attention.

### 3.2. The Direct Effect on Female Reproductive Function

In recent years, ICIs have become part of the standard treatment regimen for women with advanced breast cancer, as well as female pediatric patients with relapsed or refractory classical Hodgkin’s lymphoma. Preserving endocrine ovarian function and the future ovarian reserve are central considerations in the management of both prepubertal and premenopausal patients. Therefore, it is crucial to determine whether ICIs have an impact on ovarian function and long-term fertility potential in order to provide informed oncofertility counseling [26].

Even in the metastatic disease, it is essential to inform patients about potential risks of hypogonadism or infertility, given that a subset of individuals may achieve sustained complete remission and subsequently contemplate future parenthood [26].

Currently, no published clinical data exist on the direct impact of ICIs on the ovarian function in female pediatric or adult patients. The available literature is limited to preclinical studies involving animal experiments aimed at understanding their mechanism of action. In a preclinical study by Xu et al., prepubertal immunocompetent and immunodeficient female mice were administered pembrolizumab or anti-mouse PD-1 antibody. A significant reduction in the number of primordial follicles was observed in immunocompetent mice following administration of both pembrolizumab and an anti-mouse PD-1 antibody. In contrast, no significant changes in follicle counts were noted in immunodeficient (nude) mice. Furthermore, superovulation assays and assessments of pubertal onset, such as vaginal opening, showed no significant differences in the number of cumulus–oocyte complexes (COCs) or in the timing of puberty between control and treatment groups, suggesting that short-term fertility remains unaffected. Notably, upregulation of cyclooxygenase-2 (COX-2) and a subsequent increase in the pro-inflammatory cytokine TNF-α were detected in ovarian tissue. Additionally, infiltration of CD3^+^ T cells was identified within ovarian follicles and in the stromal compartments of ovaries treated with the anti-mouse PD-1 antibody. These findings suggest that PD-1 immune checkpoint blockade may compromise the ovarian reserve through an inflammatory pathway involving CD3^+^ T-cell infiltration [27].

In another preclinical study, investigators used both tumor-bearing and tumor-free adult female mouse models to assess the impact of PD-L1 and CTLA-4 blockade on the ovarian function [28]. The results offer clear evidence of how immune checkpoint inhibitor therapy affects ovarian function. The study also highlights how immune cells and the cytokines they release regulate normal ovarian activity. Through a detailed assessment involving multiple ex vivo and in vivo models, it was demonstrated that an increase in ovarian immune cells and inflammatory cytokines can induce follicular atresia. This effect is observed across all stages of follicular development, ranging from immature primordial follicles to fully matured ovulated oocytes [28].

In non-human primate models, checkpoint antibodies have been shown to bind to ovarian connective tissue without producing obvious histological follicular destruction [15]. While reassuring at first glance, this observation does not exclude subtle functional impairment at the molecular or endocrine level, which may escape routine histopathological detection.

From a clinical perspective, premature ovarian insufficiency represents the most severe manifestation of female gonadal toxicity. Defined by hypergonadotropic hypogonadism before the age of 40, POI is characterized by amenorrhea, hypoestrogenism, and elevated gonadotropin levels with profound implications for fertility, bone health, cardiovascular risk, and quality of life [12].

Although still considered rare, cases of ovarian failure following ICI exposure have been reported, often in conjunction with other immune-related endocrine toxicities. Given the absence of systematic ovarian reserve monitoring in most oncology protocols, it is likely that subclinical or partial ovarian dysfunction remains underrecognized.

Menstrual irregularities, including oligomenorrhea and secondary amenorrhea, may represent early indicators of evolving ovarian impairment. However, such changes are frequently attributed to chemotherapy, stress, weight fluctuations, or hypothalamic suppression, further complicating attribution to ICIs.

In TNBC, ICIs are rarely administered as monotherapy in curative settings and are most often combined with cytotoxic chemotherapy. This dual exposure raises the possibility of additive gonadotoxic effects, where both treatments independently contribute to follicular loss, or synergistic toxicity, whereby immune-mediated inflammation amplifies chemotherapy-induced ovarian damage.

Chemotherapy primarily targets dividing granulosa cells and growing follicles, whereas immune-mediated injury is thought to preferentially affect the primordial follicle pool and stromal immune microenvironment. Together, these mechanisms may result in more profound and less reversible ovarian reserve depletion than expected from chemotherapy alone.

This interaction is of particular clinical relevance in young women with TNBC, where neoadjuvant or adjuvant chemoimmunotherapy is now standard practice in high-risk disease.

## 4. Secondary Hypogonadism and Pituitary Axis Disruption

In addition to direct immune-mediated gonadal injury, ICIs can impair any organ system [29]. Adverse reactions usually occur in the skin, gastrointestinal tract, liver, and endocrine system [30,31]. The highest number of reports was linked to anti-PD-1 monotherapy, followed by combination regimens involving anti-CTLA-4 and anti-PD-1/PD-L1. Furthermore, the analysis indicated a male predominance among reported ICI-related with a male-to-female ratio of approximately 1.5:1. This may, in part, be attributed to the higher incidence of melanoma and non-small cell lung cancer, the two most common malignancies treated with ICIs, among male patients [32].

Secondary hypogonadism, resulting from pituitary or hypothalamic dysfunction, represents a clinically significant but frequently underrecognized consequence of immune-related endocrine toxicity. Hypophysitis is one of the most characteristic endocrine immune-related adverse events associated with ICIs, particularly during treatment with anti-CTLA4 and the combination anti-CTLA4/anti-PD-1 therapy [13,33]. Secondary hypogonadism frequently arises in the setting of panhypopituitarism, which is also characterized by secondary hypothyroidism and secondary adrenal insufficiency [34,35]. Although current literature has mainly focused on primarily adrenal insufficiency and hypothyroidism due to their potentially life-threatening consequences, the implications for the reproductive function should not be overlooked—particularly in patients with potentially curable diseases or those achieving durable remission.

Compared to hypophysitis, hypogonadism is reported as a relatively rare immune-related adverse event [13,36]. However, the distinction between primary and secondary hypogonadism remains poorly defined, and its true incidence may be underestimated due to the lack of routine assessment of sex hormones, including follicle-stimulating hormone (FSH), luteinizing hormone (LH), testosterone, and estradiol [13,36]. This diagnostic gap evaluation is clinically relevant, as isolated hypogonadotropic hypogonadism may occur even in the absence of disruptions in other pituitary axes, such as the pituitary–thyroid or pituitary–adrenal pathways. Thus, secondary hypogonadism may occur independently of panhypopituitarism, posing diagnostic challenges.

The frequency of isolated hypogonadotropic hypogonadism in patients undergoing immune checkpoint inhibitors remains unknown and requires further investigation. A prospective study involving 22 patients diagnosed with renal cell carcinoma, treated with anti-PD1, showed an increase in E2, LH/FSH ratio and a decrease in FSH in males but not in females [37].

These findings underscore the need for further research to clarify the prevalence, pathophysiology, and clinical implications of ICI-induced disruption of the pituitary–gonadal axis. Moreover, the long-term effects of immunotherapy-related hypopituitarism on fertility remain largely unknown and require systematic evaluation.

## 5. Pregnancy During and After ICI Exposure

ICIs are contraindicated during pregnancy due to their mechanism of action and potential to disrupt maternal immune tolerance toward the fetus. Preclinical studies demonstrate that PD-1/PD-L1 interactions play a crucial role in maintaining fetomaternal immune tolerance. Disruption of this pathway may increase the risk of spontaneous miscarriage, fetal growth restriction, and immune-mediated fetal complications.

Clinical data on pregnancy after ICI exposure remain extremely limited and largely consist of isolated case reports and small series [38]. Key unresolved questions include the optimal washout interval between ICI discontinuation and conception, the long-term safety of prior immune activation on placentation and fetal development, and the potential transgenerational effects of immune modulation.

Current expert opinion favors a conservative delay of several months after ICI discontinuation before attempting conception, although evidence-based thresholds are lacking.

### Clinical Case Illustration—Premature Ovarian Insufficiency After Immunotherapy in a Young Patient with a Triple-Negative Breast Cancer

The case of a 34-year-old nulliparous woman with hypothyroidism under stable substitution therapy, admitted in our clinic for a painful left breast lump identified 1 month prior to admission, is hereby reported. The patient had a significant family history of ovarian cancer involving multiple maternal relatives: mother, grandmother and aunt. Upon admission, the clinical examination revealed a palpable mass in the left upper outer quadrant (UOQ) measuring approximately 4.5 cm, adherent to the major pectoralis muscle along with two enlarged axillary lymph nodes relatively fixed to the surrounding tissue sized 2.5 cm.

The breast ultrasonography demonstrated a hypoechoic, polylobulated, hypervascularized lesion at 2 o’clock, with irregular margins and high elastographic score 4, that has a diameter of 4 cm, along with another hypervascularized, rigid on elastography, 7 mm tumor, suggestive of intraductal proliferation located 4 cm from the nipple. Two enlarged left axillary lymph nodes that showed hilum, thickened cortex (up to 5 mm compared to 2 mm on the contralateral side) were also identified.

Mammography confirmed a 3.5 cm mass with amorphous microcalcifications and three enlarged axillary lymph nodes (≥12 mm) in the left UOQ.

**Figure 1:**
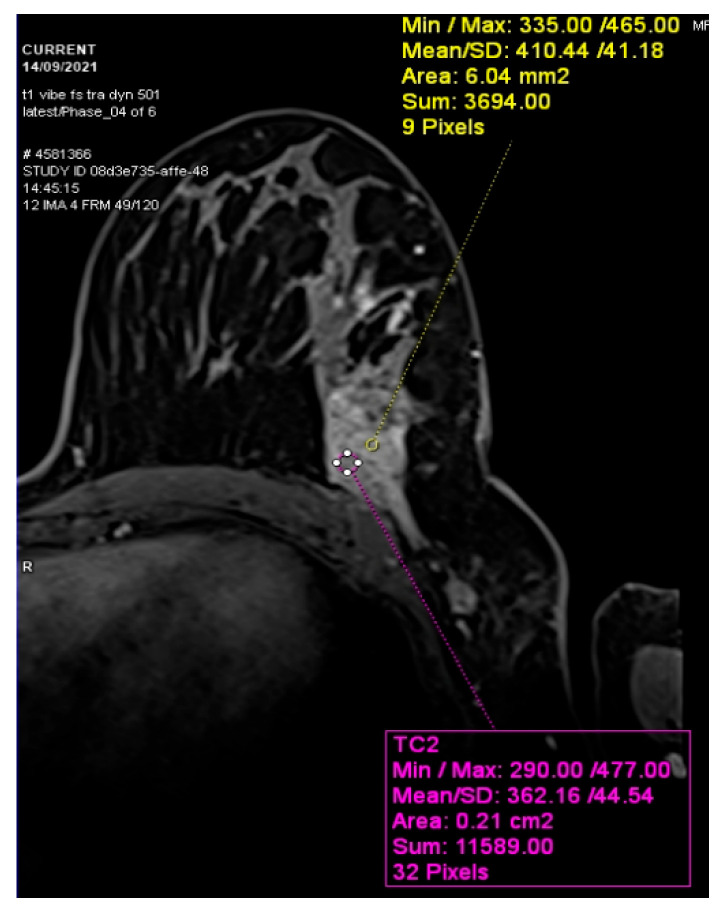
Left breast tumor MRI aspect (institutional patient data).

The contrast-enhanced (C.E) breast magnetic resonance imaging (MRI) identified the presence of both breast tumors T1 = 49/24 mm and T2 = 9 mm and multiple enlarged axillary lymph nodes of ≥14 mm (Figure 1).

Excisional breast biopsy revealed an invasive ductal carcinoma, Nottingham histologic grade III. Immunohistochemistry tests showed that the tumor was ER-negative (estrogen receptors), PR < 1% (progesterone receptors), HER2 = 1+ (negative), a Ki-67 proliferation index > 70% tumor cells, high tumor cellularity, and marked tumor-infiltrating lymphocytes, with positive PD-L1 expression.

Excisional left axillary biopsy confirmed metastasis of invasive ductal carcinoma.

**Figure 2:**
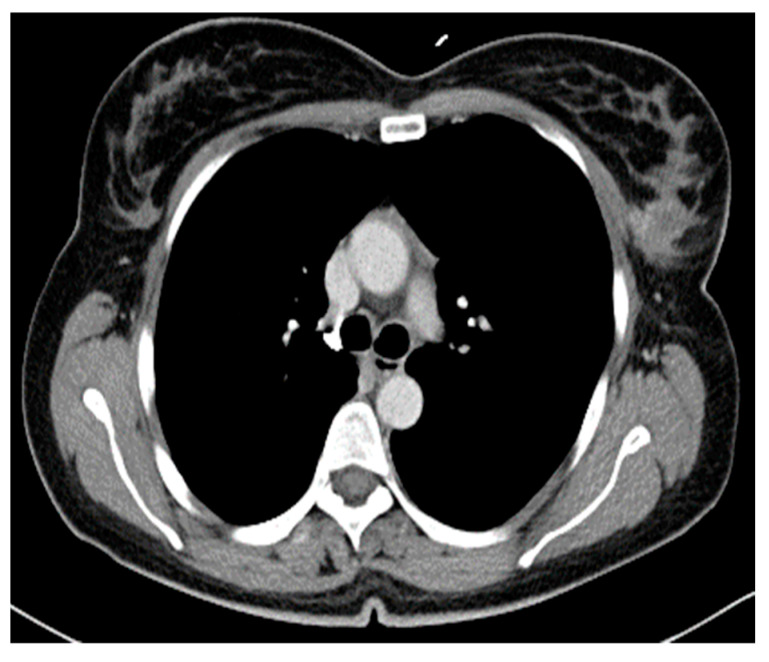
Left breast tumor CT aspect (institutional patient data).

Staging with thoraco-abdomino-pelvic computed tomography showed that the larger left breast tumor located in the UOQ had a possible invasion of the pectoralis major muscle and multiple enlarged left axillary lymph nodes, suspicious left subclavian lymph nodes, but also a hepatic tumor sized 15/17 mm with characteristics of a hepatic adenoma or hemangioma with no identifiable metastasis (Figure 2).

**Figure 3:**
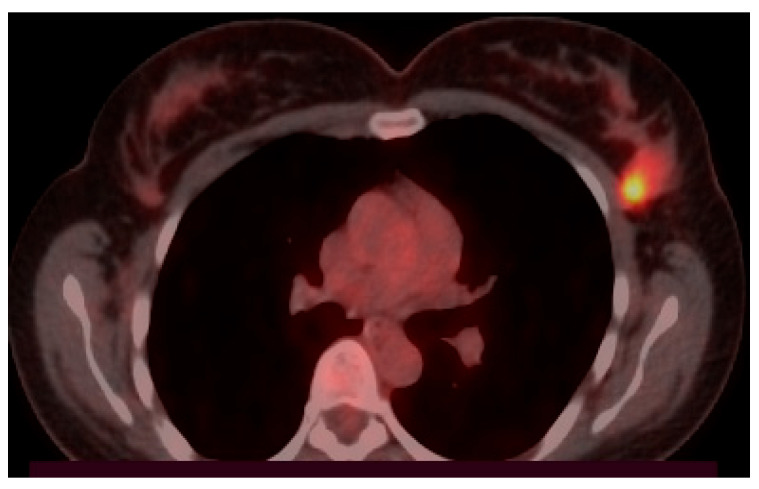
Metabolic active tumor on the left breast PET-CT aspect (institutional patient data).

Positron Emission Tomography/Computed Tomography (PET-CT) with fluorodeoxyglucose F-18 confirms the presence of the metabolic active tumor in the UOQ of the left breast and the left axillary lymph nodes, but with no other metabolically active lesions (Figure 3).

Taking into consideration the family history, the patient underwent genetic testing that showed BRCA1 gene mutation. The patient agreed for bilateral mastectomy with immediate reconstruction and bilateral oophorectomy after childbirth completion.

Given the fact that the patient is nulliparous, oncofertility counseling was performed, but the patient declined the opportunity for oocytes or embryos cryopreservation. The patient consented only to concurrent ovarian suppression using a gonadotropin-releasing hormone (GnRH) agonist during systemic cytotoxic therapy. Baseline transvaginal ultrasound showed a normal ovarian reserve, about 15 antral follicles; however, anti-Mullerian hormone (AMH) was not assessed at that time.

The patient was enrolled in an institutional clinical study and received atezolizumab in combination with nab-paclitaxel, with the first dose administered as part of the study protocol. The study was approved by the institutional ethics committee, and written informed consent was obtained from the patient. Every week, for a period of 14 weeks, the patient reported to the clinic for treatment, as the size of the left breast tumor and ipsilateral axillary lymph nodes, biological constants and possible adverse treatment effects were being evaluated.

**Figure 4:**
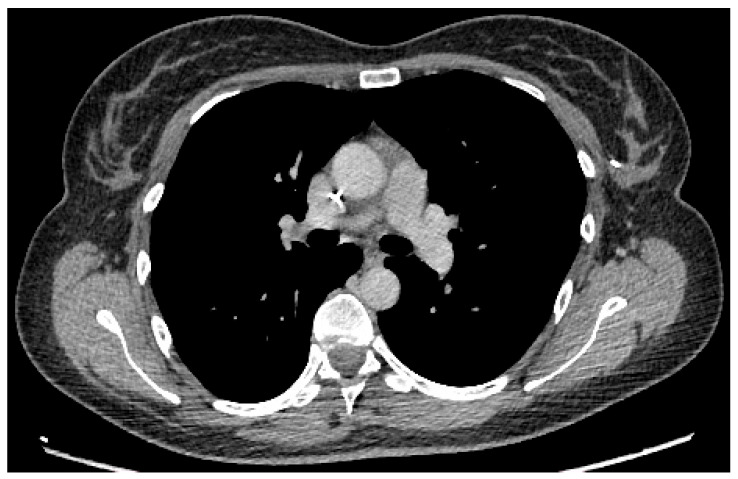
Two months of therapy CT scan control (institutional patient data).

After approximately 2 months of therapy, imaging demonstrated a marked partial response with near-complete radiological regression of the primary tumor on the left, axillary lymphadenopathy with short axis < 15 mm (significantly unchanged) and suspicious left subclavian lymph nodes (significantly unchanged) (Figure 4).

The patient was monitored oncologically during the 14 weeks of treatment. She was admitted to our service for a double mastectomy with immediate reconstruction: a left modified radical mastectomy and a right prophylactic mastectomy. Postoperative imaging confirmed complete radiological response.

**Figure 5:**
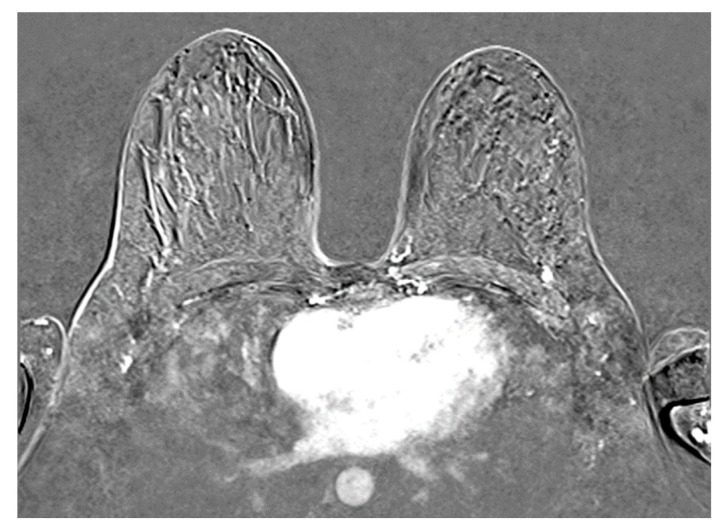
Post treatment control MRI (institutional patient data).

**Figure 6:**
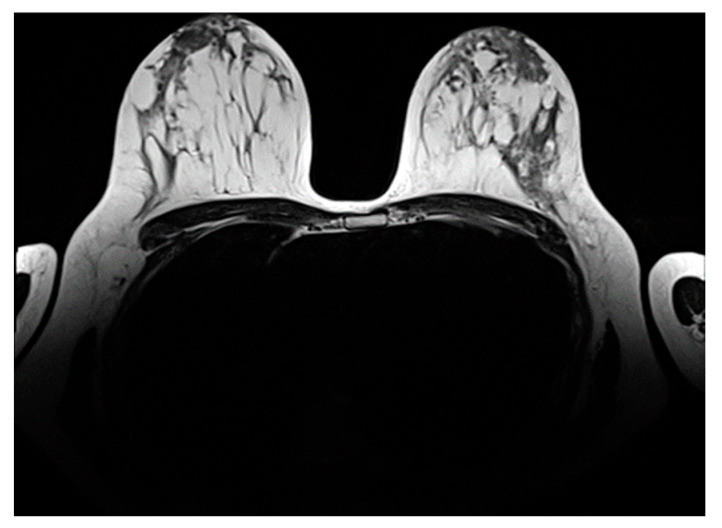
Post treatment control MRI T2 (institutional patient data).

Contrast breast MRI showed an invisible breast tumor (Figure 5 and Figure 6).

After finishing this stage of treatment, the patient was proposed adjuvant chemotherapy and PARP inhibitors, but the patient agreed only to 3 cycles of Epirubicin-Cyclophosphamide (EC). After finishing the 3 cycles of EC, a treatment with Olaparib was followed.

The oncological treatment ended after 29 sessions of external radiotherapy for the left breast and the left axile. The last session was on 15 July 2022.

Since then, she has remained in complete oncological remission and continues regular follow-up. Two years after the completion of systemic therapy, the patient expressed a desire for spontaneous pregnancy. Reproductive evaluation revealed a severely compromised ovarian reserve, with an antral follicle count of four and an AMH level of 0.16 ng/mL (unit standard), consistent with premature ovarian insufficiency. The patient continues to pursue natural conception and has declined in vitro fertilization to date.

This case illustrates a profound and persistent decline in ovarian reserve following combined chemoimmunotherapy including atezolizumab in a young woman with TNBC. While the contribution of chemotherapy cannot be excluded, the severity of ovarian failure despite GnRH analog co-treatment, the patient’s young age at exposure, and the preclinical evidence of immune-mediated ovarian injury support the hypothesis that immunotherapy may have played an additive role in accelerating reproductive aging.

This case should not be interpreted as evidence of causality but rather as a clinically relevant illustration of the complex interaction between chemotherapy, immunotherapy, and reproductive aging.

## 6. Discussion

Breast cancer remains a public health issue for women. Thanks to constant standardization and progress in medicine, the long-term prognosis has significantly improved for a patient undergoing standard screening protocols. However, an important increase in survival rates and long-life expectancy for neoplastic patients exposes them to the long-term effects of anti-neoplastic therapies that can, in some cases radically, alter the survivorship experience. Nowadays, the goal of medicine is to ensure equal quality of life and living standards for its patients, besides curability. Despite their importance, fertility and other ovarian function measures are not usual endpoints in breast cancer clinical trials. Ovarian toxicity is a recognized adverse effect of chemotherapy for premenopausal women, but for ICIs, in contrast to the vast body of evidence regarding their clinical utility, there is a paucity of data about any detrimental effect on fertility, future pregnancies, or sexuality.

This review highlights the emerging and still insufficiently characterized issue of reproductive toxicity associated with ICIs in patients with TNBC. The data hereby synthesized converge toward the concept that reproductive dysfunction in the context of ICI therapy may arise through dual mechanisms: direct immune-mediated gonadal injury and indirect disruption of the hypothalamic-pituitary-gonadal axis.

Preclinical evidence strongly supports the biological plausibility of direct immune-related ovarian and testicular damage. Experimental models consistently demonstrate immune cell infiltration of gonadal tissue, cytokine-driven inflammatory cascades, follicular atresia, and impaired spermatogenesis after PD-1/PD-L1 and CTLA-4 blockade. Unlike chemotherapy, which preferentially targets rapidly dividing germ cells, ICIs interfere with immune tolerance within the gonadal microenvironment, potentially affecting both germ cells and supporting endocrine and stromal compartments. This distinction may explain the delayed, progressive, and sometimes irreversible nature of gonadal failure reported in some patients long after treatment completion.

Clinical evidence, although still limited, aligns with these mechanistic observations. Reports of immune-mediated orchitis, epididymo-orchitis, azoospermia, and testosterone deficiency in men, as well as emerging descriptions of ovarian dysfunction and premature ovarian insufficiency in women, suggest that immune-related reproductive toxicity represents a genuine clinical entity rather than a theoretical concern. However, the true incidence remains unknown because of the absence of systematic baseline and longitudinal reproductive monitoring in clinical trials and routine oncology practice.

The clinical case presented in this work provides a compelling illustration of these mechanisms in a young woman with TNBC who developed severe ovarian reserve depletion and premature ovarian insufficiency after chemoimmunotherapy, including atezolizumab. While a causal relationship cannot be definitively established, the temporal association, the profound decline in ovarian reserve, and the preclinical data supporting immune-mediated follicular loss strongly suggest a contributory role of immunotherapy. Importantly, this case also emphasizes the potential additive or synergistic gonadotoxic effects of chemotherapy combined with ICIs, a scenario that is now a standard in the neoadjuvant and adjuvant treatment of high-risk TNBC.

From a clinical standpoint, this dual mechanism complicates the assessment of fertility risk. Chemotherapy-related gonadotoxicity is relatively predictable and age-dependent, whereas ICI-related immune toxicity remains unpredictable, potentially delayed, and poorly quantified. Consequently, the traditional fertility counseling models developed for chemotherapy alone are no longer sufficient for patients receiving modern chemoimmunotherapy regimens. This uncertainty must be explicitly communicated to patients during shared decision-making.

The implications for oncofertility practice are substantial. Early fertility counseling should be systematically offered to all young patients with TNBC prior to initiating immunotherapy-containing regimens, regardless of baseline ovarian reserve or perceived low risk. Oocyte or embryo cryopreservation remains the most reliable fertility preservation strategy, while GnRH analogs may offer partial protection against chemotherapy-induced damage but cannot be assumed to prevent immune-mediated ovarian injury. The present case further illustrates that ovarian suppression alone may be insufficient to prevent severe long-term reproductive sequelae in the setting of combined chemoimmunotherapy.

Secondary hypogonadism resulting from immune-mediated hypophysitis represents an additional and often underdiagnosed contributor to reproductive dysfunction. Since isolated pituitary–gonadal axis impairment may occur without overt adrenal or thyroid failure, many cases likely remain unrecognized. This highlights the urgent need for routine integration of gonadotropin and sex steroid monitoring into endocrine surveillance protocols for patients receiving ICIs, particularly in younger age groups.

Beyond fertility itself, the long-term health consequences of hypogonadism, including osteoporosis, cardiovascular risk, sexual dysfunction, and psychological distress, must be considered integral components of survivorship care. These issues are particularly impactful in young cancer survivors, for whom endocrine and reproductive sequelae may persist for decades.

Several important limitations must be acknowledged. First, the current literature is dominated by preclinical studies, retrospective analyses, and isolated case reports, precluding robust risk quantification. Second, the specific contribution of ICIs versus chemotherapy to gonadal injury cannot be reliably disentangled in most clinical settings. Third, predictive biomarkers for immune-mediated reproductive toxicity are lacking, preventing individualized risk stratification. Fourth, these findings must be interpreted in light of the standard use of combined chemoimmunotherapy in TNBC, which precludes definitive attribution of ovarian toxicity to immune checkpoint inhibitors alone.

Finally, pregnancy outcomes after ICI exposure remain poorly documented, and long-term transgenerational safety data are virtually nonexistent.

Despite these limitations, the available evidence already justifies a precautionary, proactive approach to reproductive health in patients with TNBC treated with immunotherapy. Multidisciplinary oncofertility pathways, involving oncologists, endocrinologists, reproductive specialists, and psycho-oncology teams, should become the standard of care. Structured clinical registries and prospective studies focused on reproductive and endocrine endpoints are urgently needed to transform current expert-based recommendations into evidence-based guidelines.

In this evolving therapeutic era, the success of immunotherapy should no longer be measured solely by tumor response and survival, but also by the preservation of long-term endocrine function, reproductive potential, and quality of life.

### Future Research Direction

Despite increasing awareness of potential reproductive and endocrine effects associated with immune checkpoint inhibitors, substantial knowledge gaps remain. Addressing these gaps will require coordinated and methodologically robust research efforts.

First, the establishment of prospective registries specifically capturing reproductive and endocrine outcomes in patients receiving immunotherapy is essential. Such registries should include standardized baseline assessments of ovarian reserve and longitudinal follow-up to better characterize the incidence, timing, and reversibility of ovarian dysfunction.

Second, there is a clear need for standardized fertility and endocrine endpoints in immunotherapy clinical trials. Incorporating reproductive outcomes, menstrual function, and hormonal markers into trial design would allow systematic evaluation of gonadal effects and facilitate cross-study comparisons.

Third, biomarker development represents a critical research priority. Identification of predictive markers of immune-mediated gonadal injury such as immunologic, genetic, or inflammatory biomarkers could enable risk stratification and tailored counseling for patients undergoing immunotherapy.

Finally, the implementation of structured endocrine monitoring protocols before, during, and after immune checkpoint inhibitor therapy warrants further investigation. Defining optimal monitoring strategies may improve early detection of endocrine dysfunction and support timely intervention in young patients treated with curative intent.

## 7. Conclusions

Immune checkpoint inhibitors have profoundly transformed the therapeutic landscape of triple-negative breast cancer, offering meaningful survival benefits in both early-stage and metastatic diseases. As the use of immunotherapy continues to expand into curative settings, survivorship issues—particularly those related to endocrine function, fertility, and long-term quality of life—have become increasingly relevant. In this context, the potential reproductive toxicity of ICIs represents an emerging and clinically important challenge.

Although definitive clinical evidence remains limited, converging data from preclinical models, pharmacovigilance reports, and small clinical series indicate that ICIs may impair reproductive function through both direct immune-mediated gonadal injury and indirect disruption of the hypothalamic–pituitary–gonadal axis. These mechanisms differ fundamentally from classical chemotherapy-induced gonadotoxicity and may lead to delayed, unpredictable, and sometimes irreversible reproductive consequences. Male patients may experience impaired spermatogenesis, testosterone deficiency, or azoospermia, while female patients may face diminished ovarian reserve, premature ovarian insufficiency, and endocrine-related infertility.

In young women with TNBC who often have a long-life expectancy after a successful treatment, these risks acquire particular clinical and psychosocial significance. However, the absence of prospective reproductive monitoring, predictive biomarkers, and standardized fertility endpoints in immunotherapy trials severely limits the ability to quantify risk and provide evidence-based counseling.

Until robust clinical data become available, a precautionary, patient-centered approach is warranted. Early fertility counseling, timely access to fertility preservation strategies, baseline and longitudinal endocrine surveillance, and multidisciplinary survivorship care should be systematically integrated into the management of reproductive-age patients receiving ICIs.

In conclusion, while immune checkpoint inhibitors represent a major therapeutic advance in TNBC, their potential long-term impact on reproductive health must no longer be considered a peripheral issue. Addressing this gap through dedicated research, structured registries, and integrated oncofertility pathways is essential to ensure that long-term survival is matched by preserved reproductive potential and quality of life.

## Figures and Tables

**Table 1:** Current recommendations related to immune checkpoint inhibitor–associated reproductive outcomes.

Guideline	Fertility Counseling Recommended	Chemotherapy-Related Gonadotoxicity Addressed	Immunotherapy-Specific Guidance	Endocrine Monitoring	Identified Gaps
ASCO	Yes	Yes	No	Not specified	Lack of immunotherapy-specific fertility and endocrine recommendations
ESMO	Yes	Yes	No	Not specified	No standardized reproductive endpoints for immune checkpoint inhibitors
ESHRE	Yes	Yes	No	Limited	Immune-mediated gonadal toxicity not addressed

Abbreviations: ASCO, American Society of Clinical Oncology; ESMO, European Society for Medical Oncology; ESHRE, European Society of Human Reproduction and Embryology.

## Data Availability

The data presented in this study are available upon reasonable request from the corresponding author. Due to the inclusion of sensitive patient-related information, the data are not publicly available in order to protect patient privacy and confidentiality. Furthermore, this clinical case has not been previously published in any medical journal, nor has it been presented at any medical conference or scientific meeting.
